# Effect of adverse events on non-adherence and study non-completion in malaria chemoprevention during pregnancy trial: A nested case control study

**DOI:** 10.1371/journal.pone.0262797

**Published:** 2022-01-19

**Authors:** Noel Patson, Mavuto Mukaka, Ingrid Peterson, Titus Divala, Lawrence Kazembe, Don Mathanga, Miriam K. Laufer, Tobias Chirwa

**Affiliations:** 1 School of Public Health, University of the Witwatersrand, Johannesburg, South Africa; 2 School of Public Health and Family Medicine, College of Medicine, University of Malawi, Blantyre, Malawi; 3 Mahidol Oxford Tropical Medicine Research Unit (MORU), Bangkok, Thailand; 4 Centre for Tropical Medicine, Nuffield Department of Medicine, University of Oxford, Oxford, United Kingdom; 5 Center for Vaccine Development and Global Health, University of Maryland School of Medicine, Baltimore, MD, United States of America; 6 TB Centre, London School of Hygiene and Tropical Medicine, London, United Kingdom; 7 Helse Nord Tuberculosis Initiative, College of Medicine, University of Malawi, Blantyre, Malawi; 8 Department of Biostatistics, University of Namibia, Windhoek, Namibia; Rabin Medical Center, Beilinson Hospital, ISRAEL

## Abstract

**Background:**

In drug trials, adverse events (AEs) burden can induce treatment non-adherence or discontinuation. The non-adherence and discontinuation induce selection bias, affecting drug safety interpretation. Nested case-control (NCC) study can efficiently quantify the impact of the AEs, although choice of sampling approach is challenging. We investigated whether NCC study with incidence density sampling is more efficient than NCC with path sampling under conditional logistic or weighted Cox models in assessing the effect of AEs on treatment non-adherence and participation in preventive antimalarial drug during pregnancy trial.

**Methods:**

Using data from a trial of medication to prevent malaria in pregnancy that randomized 600 women to receive chloroquine or sulfadoxine-pyrimethamine during pregnancy, we conducted a NCC study assessing the role of prospectively collected AEs, as exposure of interest, on treatment non-adherence and study non-completion. We compared estimates from NCC study with incidence density against those from NCC with path sampling under conditional logistic and weighted Cox models.

**Results:**

Out of 599 women with the outcomes of interest, 474 (79%) experienced at least one AE before delivery. For conditional logistic model, the hazard ratio for the effect of AE occurrence on treatment non-adherence was 0.70 (95% CI: 0.42, 1.17; p = 0.175) under incidence density sampling and 0.68 (95% CI: 0.41, 1.13; p = 0.137) for path sampling. For study non-completion, the hazard ratio was 1.02 (95% CI: 0.56, 1.83; p = 0.955) under incidence density sampling and 0.85 (95% CI: 0.45, 1.60; p = 0.619) under path sampling. We obtained similar hazard ratios and standard errors under incidence density sampling and path sampling whether weighted Cox or conditional logistic models were used.

**Conclusion:**

NCC with incidence density sampling and NCC with path sampling are practically similar in efficiency whether conditional logistic or weighted Cox analytical methods although path sampling uses more unique controls to achieve the similar estimates.

**Trial registration:**

ClinicalTrials.gov: NCT01443130.

## Introduction

Treatment non-adherence and treatment discontinuation, in which patients either do not take their medication as prescribed or do not take it at all, is a challenge in analysis and interpretation of randomized controlled trials results since it can lead to underestimation of treatment effects [1]. Adverse events (AEs) occurrence is one of the factors that influence treatment non-adherence and discontinuation. Therefore, treatment non-adherence and discontinuation can be considered as proxy outcomes for a measure of the impact of AEs on patients in clinical trials [2]. However, most trials descriptively report treatment non-adherence and discontinuation information in relation to AEs under safety and tolerability section and participant flow chart of the main study report [3–5]. Such kind of reporting and analysis is less informative since it does not provide complete estimates on how the AEs affect non-adherence over the follow-up time, after accounting for potential confounders e.g. patient age. Further, AEs that impact treatment non-adherence and study discontinuation outcomes precede these outcomes. Ignoring the fact that the AEs that impact treatment non-adherence and study non-completion outcomes occur before the observation of the treatment non-adherence or discontinuation can lead to underestimation of the effect of AEs [6, 7]. Establishing association between the AEs and treatment non-adherence or discontinuation is also both statistically and ethically challenging since the AE exposure cannot be randomized and it is difficult to pre-specify at design stage. Use of a nested case-control approach to understand the impact of AEs can be useful in this context since it derives a sample of cases and controls that are comparable, accounting for potential differences in follow-up time. However, the choice of an efficient nested case-control design and analytical method can present challenges. In order to address this challenge, empirical comparison of competing nested case-control designs is useful especially towards the choice of the most efficient nested case-control sampling and analytical approach.

Traditionally incidence density sampling (i.e. risk-set sampling) is used in most nested case-control studies. Under a nested case-control study with incidence density sampling design, each index case is matched to a specified number of controls sampled at the time of index case occurrence. The nested case-control study with incidence density sampling design is considered as an unbiased and statistically efficient approach to cohort sampling that accommodates causal interpretation of the observed effect of an exposure on an outcome [8]. The matching on time ensures that the cases are comparable to control by minimizing confounding that would arise due to the differences in follow-up time between the cases and controls. Hence, the obtained estimates are unbiased and reflect the original trial cohort [9]. The selection of controls is done with replacement such that a subject could be selected as a control multiple times and may later become a case. However, it has also been argued that greater efficiency can also be achieved if the set of controls for different cases are as disjoint as possible. For instance, Langholz and Thomas proposed a modified alternative sampling approach that aims at ensuring that minimum number of controls are used more than once [10]. Under this second alternative nested case-control sampling approach, firstly a path set is constructed that includes all subjects who entered the study before *t*_*i*_ in time interval (*t*_*i*−1_, *t*_*i*_) and exited after time *t*_*j*_ in time interval (*t*_*j*_, *t*_*j*+1_). Under this second sampling approach the specified number of controls are randomly sampled from the path set without replacement until all the controls in that path set are selected and the path set can be replenished. Once the desired number of controls is achieved the path sampling is stopped. As defined elsewhere, in this paper we refer to this variation of nested case-control with incidence density sampling design as nested case-control study with *path sampling* design [11]. Based on the provided descriptions of the nested case-control with incidence density sampling and nested case-control with path sampling, path sampling samples more unique patients than the traditional incidence density sampling. In practice, it is important to choose a nested case-control sampling design that can optimally estimate the effect of AEs on treatment non-adherence and study non-completion. However, there is limited work highlighting whether inclusion of more unique controls via path sampling offers an added advantage over incidence density sampling.

Conventionally, conditional logistic regression or Cox regression stratified on case-control sets are used in analysis of most nested case-control studies [12]. Recently inverse probability weighting (IPW) Cox models have been shown to have higher power and efficiency than conditional logistic regression analysing nested case-control studies [13–16]. Under IPW Cox models, individual log-likelihood is weighted by the inverse probability of being included in the nested case-control study. The weighting is can even offer more advantages of adapting the modelling, generally, beyond proportional hazards regression models [17]. Furthermore, the weighted Cox model also breaks the match to accommodate the use of cases and controls at all-time points when they are at risk.

Most of the studies discussed above have demonstrated the utility of the nested case-control with incidence density sampling under conditional logistic regression model and IPW models such as weighted Cox regression model. However, there is limited work demonstrating the utility and comparing the efficiency of the two alternative sampling designs (i.e. incidence density sampling and path sampling) under IPW Cox models and the traditional conditional logistic regression. In this paper, we investigated whether nested case-control with path sampling is more efficient than nested case-control study with incidence density sampling under conditional logistic regression model and weighted Cox regression model. We empirically compared nested case-control with incidence density sampling design against nested case-control with path sampling design under weighted Cox model and conditional logistic regression model, in investigating the effect of AEs on treatment non-adherence or study non-completion in the context of intermittent preventive treatment of malaria in pregnancy (IPTp) trial as case study. We hypothesized that path sampling design and weighted Cox regression will yield more efficient estimates with lower standard errors and AE exposure will increase the risk of non-adherence or discontinuation.

## Methods

### Motivating data description

To assess the effect of AEs on treatment non-adherence and study non-completion, we conducted a secondary data analysis of data from a clinical trial of preventive treatment for malaria in pregnancy Malawi (NCT01443130) using nested case-control study approach. The trial was a randomised, open-label, controlled clinical trial evaluating whether chloroquine for intermittent preventive treatment to prevent malaria in pregnancy (IPTp-CQ) or chloroquine chemoprophylaxis was better than IPTp using sulfadoxine-pyrimethamine (IPTp-SP). The trial included women in first or second trimester before 27 weeks of gestational age, attending first antenatal visit and willing to remain in the study are until 14 weeks after delivery. The trial excluded women who were HIV positive, on any known chronic therapy with antimalarial or anti-folate drugs and had known high risk pregnancies.

Between February 2012 and May 2014 a total of 600 pregnant women in their first or second trimester were recruited and randomized to receive (a) sulfadoxine-pyrimethamine (SP) IPTp (two doses, four weeks apart), (b) chloroquine IPTp (600 mg on day 1, 600 mg on day 2, and 300 mg on day 3 two days apart), (see S1 Fig under supplementary materials). In the current analysis, we excluded a third arm consisting of 300 women randomized to receive weekly chloroquine prophylaxis since that arm had a radically different treatment schedule compared to the IPTp arms. The study treatments were administered under directly observed therapy. Detailed procedures, participant demographics and treatment effects for the trial are described in the primary trial manuscript [18]. The clinical trial protocol was registered with ClinicalTrials.gov (NCT01443130).

Safety and tolerability assessments were based on unsolicited adverse events collected from the first day of the treatment administration until the final study visit. AEs were graded based on a standardized protocol [19], (i) graded on a four-point scale as, mild, moderate, severe, or life-threatening and (ii) further classified by System Organ Class category. The clinical trial was reviewed and approved by Institutional Review Board at the University of Maryland, the College of Medicine Research and Ethics Committee at the University of Malawi and the Malawi Pharmacy Medicines and Poisons Board. From every participant, a written informed consent was obtained just before undergoing any trial procedure. During the current analysis we only considered follow-up time from enrolment up to delivery because medication was administered until delivery.

### Nested case-control study designs

We considered two types of nested case-control study designs; (a) Nested case-control study with risk-set sampling (i.e. incidence density sampling) (b) Nested case-control study with path sampling. The samples for the two designs were derived from the cohort *ς* = {*i*,……,*n*} of women who had taken at least their first dose of IPTp administered in the trial. Both nested case-control designs were based on two study outcomes: (i) treatment non-adherence and (ii) study non-completion. However, if the only outcome of interest (e.g. treatment non-adherence or study non-completion) occurred only at enrolment time (t = 0), the woman was considered ineligible for the nested case-control study since matching was done on follow-up time (t>0). For both designs we identified same set of baseline characteristics as potential confounders. The potential confounders included maternal age in years, body mass index (BMI; calculated as weight in kilograms divided by height in metres squared), gravidity (primigravid or not) and, IPTp treatment arm.

#### Nested case-control with incidence density sampling

Definition of a case was based on experiencing an outcome of interest as defined in the preceding section. Firstly, we defined cases of *treatment non-adherence* as those missing at least one dose of treatment after the first dose. Although there was a possibility of observing more than one non-adherence events per woman, we focused on time to the first treatment non-adherence after enrolment. Secondly, we defined *study non-completion* as discontinuing participation in the study before delivery.

The controls were defined as women who had not yet experienced the outcome interest (i.e. *treatment non-adherence* or *study non-completion*) but were at risk to become cases. For each treatment non-adherence and study non-completion cases, we randomly selected four controls using risk set sampling (i.e. incidence density sampling). The selection of the controls (and corresponding analyses) was done separately for each endpoint. Each case was matched on follow-up time since enrolment by selecting controls from the set of women in the clinical trial cohort who had not experienced the outcome up until the time that the case arose; controls were selected without regard to outcomes that might occur after the time of matching. Hence a control could be sampled multiple times and could later become a case. We considered the selection of four controls per case as sufficient since there is negligible gain in statistical efficiency when more than four controls are selected per case [20–22].

The exposure in this analysis was having an AE before selection in risk-set sampling. We dichotomized the AEs as (having an AE versus not having one before the index case time). Due to the exploratory nature of our study in demonstrating the efficiency of incidence density sampling over path sampling under different analytical methods, we considered only any AE exposure regardless of the relatedness to the treatment or severity.

#### Nested case-control with path sampling

The definition of cases and exposure are as in the incidence density sampling above. Every aspect for the path sampling approach is similar to the standard nested case-control with incidence density sampling except that this approach (i.e. path sampling) attempted to minimize the number of times that a control could be selected more than once. This was achieved using the path set concept where we sampled a path set according to the probabilities given the path set representation in the entire risk set at each event time [10]. As highlighted in the introduction section above, Langholz and Thomas indicate that the path set *Q*_*ij*_ consist of all subjects who entered the study before *t*_*i*_ in time interval (*t*_*i*−1_, *t*_*i*_) and exited after time *t*_*j*_ in time interval (*t*_*j*_, *t*_*j*+1_). In our context, the path set consisted of women who enrolled before *t*_*i*_ and exited the trial after *t*_*j*_, in the respective time intervals. After the path set was defined and sampled, the controls were randomly sampled from the selected path set until the whole path set was emptied when the set can be replenished (i.e. once a woman was selected as a control from the path set, she could not be selected again until all women in that path set were selected). We implemented this sampling design using the recently illustrated implementation of the design in R statistical software [11]. This sampling approach yields as unique number of patients as possible included in the nested case-control study. Therefore, path sampling attempts to use more information from the controls by sampling more unique controls from the cohort. As the case with nested case-control with incidence density sampling, the selection of the controls (and corresponding analyses) was done separately for each endpoint.

### Statistical analysis

We conducted all the data management and statistical analyses in Stata version 15 (StataCorp, College Station, TX, USA) and R Statistical software packages.

#### Descriptive analysis

Firstly, we summarized the baseline characteristics of the study participants by case-control status. For continuous variables, we reported mean (standard deviation [SD]) or median (inter-quartile range [IQR]) and proportion for categorical variables. We also summarized the distribution of AE occurrence within respective treatment arms based on AE grade and frequency. We plotted Kaplan Meier graph to characterise time to first AE occurrence between the IPTp arms.

#### Modelling the effect of AEs on non-adherence and study non-completion

Let likelihood for the nested case-control for parameter *β* be *L*_*ncc*_(*β*). Traditionally, estimates in nested case-control studies are derived by maximizing partial likelihood function that can be written as

$$L_{ncc}(\beta )=\prod_{i∊\Omega }\frac{exp(\beta^{\prime }X_{i})}{\sum_{j∊{Ȓ}_{i}}exp(\beta^{\prime }X_{j})}$$ (M1)

Where ***L***_***ncc***_(***β***) is likelihood for nested case-control for parameter ***β***. The ***β*** represents a vector of the regression coefficients, such that ***β***′ is a transposed vector corresponding to vector(s) of covariates. The ***X***_***i***_ **and *X***_***j***_ are the vectors of covariates for the individuals *i* and *j*. The sampled risk set Ȓ_*i*_ consists of case *i* and sampled *m* controls matched to the case on given time t since enrolment. The ***Ω*** represents the set of all the cases. The conditional likelihood under logistic regression coincides with Cox regression partial likelihood [23]. Therefore, the analysis of nested case-control study using Cox regression model or conditional logistic stratified on case-control set yield similar results. In this study we firstly considered, conditional logistic models whose results are always the same as the traditional stratified Cox regression model (i.e. we stratified the model by case-control set indicator *ś (see the equation* ***M2*** *below))*. The baseline hazard for the stratum at follow-up time *t* was considered as h_0ś_(t).


$$h_{i}(t)=h_{0ś}(t)\;exp\;(\beta^{\prime }X_{i})$$ (M2)

As already defined, the partial likelihood that is maximized to obtain the estimate for the regression coefficient *β* is similar to **M1;**

$$L_{cox}(\beta )=\prod_{i∊\Omega }\frac{exp(\beta^{\prime }X_{i})}{\sum_{j∊R_{i}}exp(\beta^{\prime }X_{j})}$$ (M3)

Here under **M3**, *R*_*i*_ is a set of all patients who have not failed or been censored but are at risk of failure at that time (i.e. all patients who are still under observation just before time t). The traditional analysis above, under **M1** uses only the information from the sampled cases and controls such that the nested case-control study can sometimes be considered as a biased sample from the cohort. Weighting the partial likelihood with the inverse of the inclusion probability of being sampled mitigates this problem. The weighted Cox model partial likelihood can be written as

$$L_{wcox}(\beta )=\prod_{i∊\Omega }\frac{exp(\beta^{\prime }X_{i})\omega_{i}}{\sum_{j∊S_{i}}exp(\beta^{\prime }X_{j})\omega_{j}}$$ (M4)

Where ***S***_***i***_ is the set of cases and controls in the nested case-control sample who are at risk at time *t*_*i*_. The Cox model obtained from the maximizing this likelihood (with inverse probability weights) to estimate the coefficient is defined as weighted Cox regression model. The weights were defined as ωj=1pj, where *p*_*j*_ is probability of a woman being in the nested case-control sample. The *p*_*j*_ = 1 for all the cases. In this paper, we used Kaplan Meier (KM) method inclusion probabilities. Inclusion probabilities defined by KM method are called design based since they estimate actual sampling of the controls [24]. The R package multipleNCC [25], was used to compute the weights.

We considered both conditional logistic model and weighted Cox model in our analysis. Overall, we fitted both the conditional logistic model (conditioned on risk sets) and weighted Cox regression to both the nested case-control sample with incidence density sampling and then to the nested case-control sample with path sampling This was done separately for non-adherence and study non-completion outcomes.

In building each model, Firstly, we fitted univariate models to assess the unadjusted effects of the covariates. Then we fitted multivariable model that included the AE exposure and IPTp treatment adjusted for similar set of confounders that were significant in the univariate analysis at 0.25. Using the likelihood ratio test, we also assessed whether inclusion of interaction between the AE occurrence and treatment arm modified the observed effect. All the point estimates from the fitted models are presented with their respective 95% confidence interval. The interpretation of all the final models was made at 0.05 significance level.

## Results

**Fig 1:**
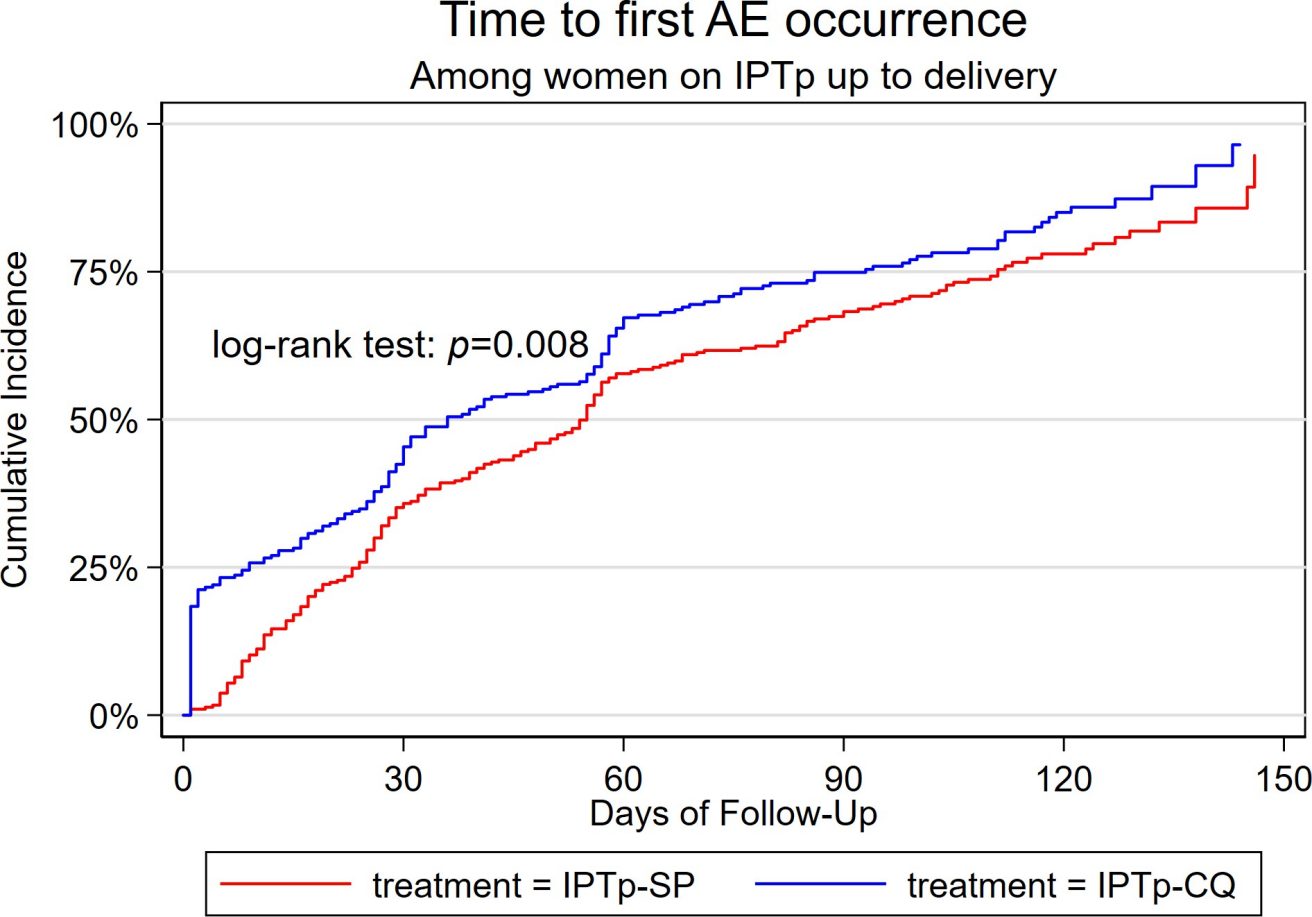
Time to first AE occurrence among the pregnant women on IPTp up to delivery.

The cohort where our nested case-control sample designs were derived considered 599 women who were assigned to the two arms of the trial. The women in the cohort had a median follow-up time of 116 days (IQR; 97, 130). At enrolment, these women had a mean gestational age of 22.1 (SD = 2.1) weeks and a mean haemoglobin concentration of 11.7 (SD = 1.3) g/dL (Table 1). A total of 1545 AEs occurred among 474 (79.0%) study participants over the follow-up time where women in the IPTp-CQ arm had a higher cumulative incidence of AEs (895 AEs) than the women in the IPTp-SP arm (650 AEs). Time to first AE was consistently higher in IPTp-CQ arm compared to IPTp-SP over the follow-up time (Fig 1) such that women in the IPTp-CQ arm were 29% more likely to experience at least one AE compared to women in the IPTp-SP arm (HR: 1.29; 95% CI: 1.07, 1.57; p = 0.008). Further details of the AE distribution are provided under supplementary S1 and S2 Tables.

**Table 1:** Distribution of the study cohort characteristics (n = 600).

Characteristic	Mean (SD) or n (%)
Maternal age(years)	21.1 (3.2)
Gestational age(weeks)	22.1 (2.1)
BMI (kg/m^2^)	23.7 (3.1)
Haemoglobin(g/dl)	11.7 (1.3)
Bed net use n (%)	450 (75.0)
Non-Primigravid n (%)	258 (43.1)
AE occurrence* n (%)	474 (79.0)
Treatment n (%)	
IPTp-SP	300 (50.0)
IPTp-CQ	300 (50.0)

For the study non-completion outcome, 64 (10.7%) women did not complete the study, with 37 (12.3%) in the IPTp-SP arm and 27(9.0%) in the IPTp-CQ arm. Based on the matching criteria defined in the methods section (matching each case to four controls), we found a total of 256 study non-completion controls. For the treatment non-adherence outcome, 106 (17.7%) women were non-adherent to trial medication, at least once during study follow-up, with 40(13.3%) in the IPTp-SP arm and 66 (22%) in the IPTp-CQ arm. After each incident case was matched to four controls, we yielded a total of 424 treatment non-adherence controls. These reported results of matching were obtained separately for the nested case-control with incidence density sampling and the nested case-control with path sampling.

### Comparison of parameter estimates

We report the hazard ratio (HR) estimates on exponentiated scale (not logarithm scale), in Tables 2 and 3. However, the standard errors are reported on logarithm scale (logHR SE), based on the corresponding log hazard ratios. The nested nested-control is abbreviated as NCC in the Tables 2 and 3. Focusing on the effect of AEs on both treatment non-adherence and study non-completion, our estimates (Tables 2 and 3) consistently indicate that path sampling yielded similar hazard ratio estimates compared to those from the incidence density sampling. Comparing the weighted Cox regression model and conditional logistic regression model, the weighted Cox model yielded similar standard errors compared to those from conditional logistic regression across both study designs and both outcomes. Therefore, overall, we obtained similar estimates of the effect of AEs on study non-completion and treatment non-adherence under both incidence density sampling and path sampling regardless of the analysis method used.

**Table 2:** Association between AE occurrence and treatment non-adherence; comparison of estimates from risk set sampling and path sampling under multivariable conditional logistic regression model and weighted Cox regression model.

	Conditional logistic regression model						Weighted Cox regression model					
	NCC with risk set sampling			NCC with Path sampling			NCC with risk set sampling			NCC with Path sampling		
Characteristic	HR (95% CI)	LogHR SE	P-value	HR (95% CI)	LogHR SE	P-value	HR (95% CI)	LogHR SE	P-value	HR (95% CI)	LogHR SE	P-value
Treatment	Reference											
*IPTp-SP*												
*IPTp-CQ*	2.07 (1.32, 3.22)	0.23	0.001	2.05 (1.30, 3.23)	0.23	0.002	2.04 (1.31, 3.16)	0.22	0.001	2.08 (1.32, 3.27)	0.23	0.002
AE occurrence	0.70 (0.42, 1.17)	0.26	0.175	0.68 (0.41, 1.13)	0.26	0.137	0.67 (0.43, 1.00)	0.22	0.051	0.68 (0.44, 1.06)	0. 23	0.087
Age	0.96 (0.89, 1.02)	0.03	0.190	0.94 (0.88, 1.01)	0.04	0.105	0.96 (0.90, 1.02)	0.03	0.179	0.95 (0.89, 1.02)	0.03	0.125

LogHR SE represents standard errors on logarithm scale of hazard ratio, NCC represents nested case-control

**Table 3:** Association between AE occurrence and study non-completion; comparison of estimates from risk set sampling and path sampling under multivariable conditional logistic regression model and weighted Cox regression model.

	Conditional logistic regression model						Weighted Cox regression model					
	NCC with risk set sampling			NCC with Path sampling			NCC with risk set sampling			NCC with Path sampling		
	HR (95% CI)	LogHR SE	P	HR (95% CI)	LogHR SE	P	HR (95% CI)	RobSE	P	HR (95% CI)	robSE	P
AE occurrence	1.02 (0.56, 1.83)	0.30	0.955	0.85 (0.45, 1.60)	0.32	0.619	0.88 (0.51, 1.52)	0.28	0.648	0.82 (0.47, 1.43)	0.29	0.477
Treatment	0.96 (0.55, 1.67)	0.28	0.896	0.68 (0.39, 1.18)	0.28	0.173	0.96 (0.55, 1.69)	0.29	0.889	0.67 (0.38, 1.18)	0.29	0.162
Age	0.92 (0.83, 1.02)	0.05	0.104	0.93 (0.84, 1.01)	0.05	0.098	0.93 (0.85, 1.01)	0.04	0.095	0.94 (0.87, 1.01)	0.04	0.072

LogHR SE represents standard errors on logarithm scale of hazard ratio, NCC represents nested case-control.

We observed no statistically significant effect of AE occurrence on treatment non-adherence under both incidence density sampling and path sampling regardless of the method of analysis used. Using conditional logistic regression, the effect of AE occurrence on treatment non-adherence hazard ratio was 0.70 (95% CI: 0.42, 1.17; p = 0.175) under incidence density sampling and under path sampling the hazard ratio was 0.68 (95% CI: 0.41, 1.13; p = 0.137). Using the weighted Cox regression, we observed a negligible departure from the overall trend where the path sampling yielded a slightly higher estimate of hazard ratio (HR: 0.68; 95% CI: 0.44, 1.06), compared to the incidence density sampling (HR: 0.67; 95% CI: 0.43, 1.00). The negligible exception could be explained as one of the expected scenarios that can arise due to sampling variation.

Similarly, we observed no effect of AE occurrence on study non-completion under both incidence density sampling and path sampling regardless of the method of analysis used (see Table 3). Using conditional logistic regression, the effect of AE occurrence on study non-completion hazard ratio was 1.02 (95% CI: 0.56, 1.83; p = 0.955) under incidence density sampling and under path sampling the hazard ratio was 0.85 (95% CI: 0.45, 1.60; p = 0.619). As expected according to the overall trend, weighted Cox regression model yielded hazard ratio of 0.88 (95% CI: 0.51, 1.52, p = 0.648) under incidence density sampling and 0.82 (95% CI: 0.47, 1.43, p = 0.477) under path sampling.

Among the adjusted potential confounders for the treatment non-adherence outcome, only treatment was associated with treatment non-adherence (Table 2). We observed two times increased risk of treatment non-adherence for patient in IPTp-CQ treatment compared those in IPTp-SP treatment. Using conditional logistic regression, the observed hazard ratio was 2.07 (95% CI: 1.32, 3.22; p = 0.001) under incidence density sampling and we observed a similar hazard ratio (HR: 2.05; 95% CI: 1.30, 3.23; p = 0.002) under path sampling. Weighted Cox regression model yielded similar hazard ratios; (HR: 2.04; 95% CI: 1.31, 3.16; p = 0.001) under incidence density sampling and (HR: 2.08; 95% CI: 1.32, 3.27; p = 0.002) under path sampling.

## Discussion

In this study, we empirically compared the efficiency of incidence density sampling and path sampling for nested case-control studies under conditional logistic regression and weighted Cox regression analysis methods. The practical application focussed on determining the impact of AEs on treatment non-adherence or discontinuation. Practically, we established that the standard errors and hazard ratios for the two designs were similar. However, it should be noted that path sampling (by design) is based on more unique controls selected [11] implying that it naturally uses a larger sample of unique patients to achieve those similar standard errors as compared to incidence density sampling. This knowledge gained is directly useful in improving nested case-control studies that have recently gained an interest in safety studies[26, 27]. This study provides insights regarding choice of nested case-control design and the burden of AEs on treatment non-adherence and study non-completion in IPTp trials. Such insights may also facilitate understanding of expected patient safety in the post-trial period when the drug is launched [28].

The negligible gain in efficiency of weighted Cox regression model over conditional regression can be attributed to the homogeneity of the cohort that we used. Since our cohort comes from a randomized clinical trial whose baseline characteristics are similar, breaking the match in order to use more controls could not add much information to our nested case-control sample that was already using over 50% of the cohort sample. Most of the substantial gains for the inverse probability weighting have been established under large sample properties [24], especially in observational studies which by design exhibit heterogeneous population unlike clinical trials.

From practical perspective, based on the example data that we used, this study did not establish a significant role of AEs on IPTp treatment non-adherence and study discontinuation. Neither, conditional logistic regression model nor the weighted Cox regression model under incidence density sampling or path sampling suggested that AE occurrence impacted treatment non-adherence or study non-completion. Our findings are contrary to the hypothesized direction of AEs effect where we postulated that AE occurrence increases the risk of IPTp non-adherence and IPTp trial discontinuation. However, our findings support what has been previously observed in IPTp context whereby frequent adverse events do not reduce adherence [29]. Our exploratory finding is useful towards ascertaining the validity of IPTp trial designs and interpretation of findings.

We observed increased risk of treatment non-adherence for the women in IPT-CQ treatment arm who were two times more likely to non-adhere compared to those in IPTp-SP arm. This is expected since the women in IPTp-CQ arm had more opportunities for non-adherence as they took two more doses compared to those on IPTp-SP arm. This agrees with previous studies that indicate that number of doses for a given regimen is inversely associated with treatment non-adherence [30]; increasing number of days and doses for taking a particular regimen can lead to low treatment adherence [31, 32]. This has consequences on the IPTp trial designs and packaging of treatment doses.

Use of the nested case-control study design in the context of clinical trial data provided a great opportunity to assess the temporal relationship of adverse events and adherence or study non-completion [33]. This further enabled us to make causal interpretations since the time-dependent AE exposure was prospectively measured and the nested case-control sampling approach reduces selection bias. Although Cox proportional hazards model with time-dependent covariates can alternatively yield similar estimates to those obtained through nested case-control approach, the nested case control is known to be more computationally efficient [34]. Despite the fact that our work may not be generalizable to settings where there are non-repeated doses, it still offers a groundwork for improved antimalarial drug safety assessment framework (accounting for non-adherence). Such improved assessment of the relationship between non-adherence to treatment and safety outcomes is crucial in planning for improved interventions effectiveness [35].

This study had some limitations. Firstly, the differences in treatment by dose schedule between the treatment arms could affect the observed differences. Secondly, we did not collect data on patient satisfaction. This made it hard it to make a conclusive statement on the unexpected observed effect of the AEs. Since non-adherence can be affected by the patient behaviour, collection of such patient satisfaction and perception in clinical trials would be helpful in elucidating the effect of AEs on non-adherence. Furthermore, the methodological conclusions in this study can be improved and extended by undertaking a full simulation study to understand the performance of the sampling and analysis methods under varying known scenarios.

## Conclusion

Assessment of safety in relation to IPTp adherence, even for less severe AEs, is an important topic in developing solutions towards improved IPTp safety profile and risk management plan in clinical trials. Nested case-control study can be considered as a useful tool in investigating the impact of AEs on treatment non-adherence and study non-completion because it offers an opportunity for an efficient sample where cases and controls are comparable, accounting for potential differences in follow-up time. We empirically demonstrated that incidence density sampling and path sampling practically yield similar estimates whether one uses weighted Cox regression or conditional logistic regression. However, by design, path sampling yields those similar estimates at the expense of more unique controls sampled compared to the incidence density sampling. The clinical findings indicated that AE occurrence did not impact treatment non-adherence or study non-completion. The insights from this study inform future trial designs and drug safety assessment. Future extension of this work can consider evaluating the impact of AEs on treatment non-adherence and study non-adherence accounting for patient perception on IPTp benefits since treatment non-adherence is associated with patient behaviour.

## Supporting information

S1 Table Distribution of SOC of AEs before delivery by treatment group. (DOCX)

S2 Table Distribution of adverse events by amongst the pregnant women on IPTp treatment. (DOCX)

S1 Fig Treatment schedule over a follow-up time for women enrolled to three malaria chemoprevention treatments in Malawi. (TIF)
