# Supplementary material for: Effect of adverse events on non-adherence and study non-completion in malaria chemoprevention during pregnancy trial: A nested case control study
Source: PLoS One. 2022 Jan 19;17(1):e0262797. doi: 10.1371/journal.pone.0262797 (PMC8769307; doi:10.1371/journal.pone.0262797)
Supplement: S1 Table — (DOCX) [file pone.0262797.s001.docx]

**S1 Table: Distribution of SOC of AEs before delivery by treatment group**

| System Organ Class of the AE | IPTp-SP n (%) | IPTp-CQ n (%) | Total |
| --- | --- | --- | --- |
| Blood and lymphatic system disorders | 9 (1.38) | 11 (1.23) | 20 (1.29) |
| Cardiac disorders | 8 (1.23) | 20 (2.23) | 28 (1.81) |
| Eye disorders | 7 (1.08) | 12 (1.34) | 19 (1.23) |
| Gastrointestinal disorders | 109 (16.77) | 204 (22.79) | 313 (20.26) |
| General disorders and administration site conditions | 44 (6.77) | 74 (8.27) | 118 (7.64) |
| Infections and infestations | 192 (29.54) | 165 (18.44) | 357 (23.11) |
| Injury, poisoning and procedural complications | 6 (0.92) | 8 (0.89) | 14 (0.91) |
| Investigations | 3 (0.46) | 6 (0.67) | 9 (0.58) |
| Metabolism and nutrition disorders | 0(0.00) | 3 (0.34) | 3 (0.19) |
| Musculoskeletal and connective tissue disorders | 75 (11.54) | 98 (10.95) | 173 (11.20) |
| Neoplasms benign, malignant and unspecified (including cysts and polyps) | 1 (0.15) | 0 (0.00) | 1 (0.06) |
| Nervous system disorders | 83 (12.77) | 149 (16.65) | 232 (15.02) |
| Pregnancy, puerperium and perinatal conditions | 51 (7.85) | 56 (6.26) | 107 (6.93) |
| Psychiatric disorders | 0 (0.00) | 1 (0.11) | 1 (0.06) |
| Renal and urinary disorders | 17 (2.62) | 27 (3.02) | 44 (2.85) |
| Reproductive system and breast disorders | 14 (2.15) | 10 (1.12) | 24 (1.55) |
| Respiratory, thoracic and mediastinal disorders | 11 (1.69) | 11 (1.23) | 22 (1.42) |
| Skin and subcutaneous tissue disorders | 5 (0.77) | 16 (1.79) | 21 (1.36) |
| Vascular disorders | 15 (2.31) | 24 (2.68) | 39 (2.52) |
| Total | 650 (100) | 895 (100) | 1545 (100) |
