# Supplementary material for: Effect of adverse events on non-adherence and study non-completion in malaria chemoprevention during pregnancy trial: A nested case control study
Source: PLoS One. 2022 Jan 19;17(1):e0262797. doi: 10.1371/journal.pone.0262797 (PMC8769307; doi:10.1371/journal.pone.0262797)
Supplement: S2 Table — (DOCX) [file pone.0262797.s002.docx]

**S2 Table: Distribution of adverse events by amongst the pregnant women on IPTp treatment**

| AE characteristic | IPTp-SP | IPTp-CQ |
| --- | --- | --- |
| Patient AE experience % (n) | 226 (75.3) | 248 (82.7) |
| Median AE frequency (IQR) | 2 (1, 3) | 2 (1, 4) |
| ^*^AE SEVERITY *% (n)* |  |  |
| 1 | 21.9 (142) | 32.3 (289) |
| 2 | 74.9 (487) | 65.3 (584) |
| ≥ 3 | 3.2 (21) | 2.5 (22) |
| *Medication-related AEs % (n) | 0.77 (5) | 23.4 (209) |

*AE severity and medication related AEs is based patients who experienced AEs (n=474)
