# Supplementary figures and images for: Effect of adverse events on non-adherence and study non-completion in malaria chemoprevention during pregnancy trial: A nested case control study
Source: PLoS One. 2022 Jan 19;17(1):e0262797. doi: 10.1371/journal.pone.0262797 (PMC8769307; doi:10.1371/journal.pone.0262797)

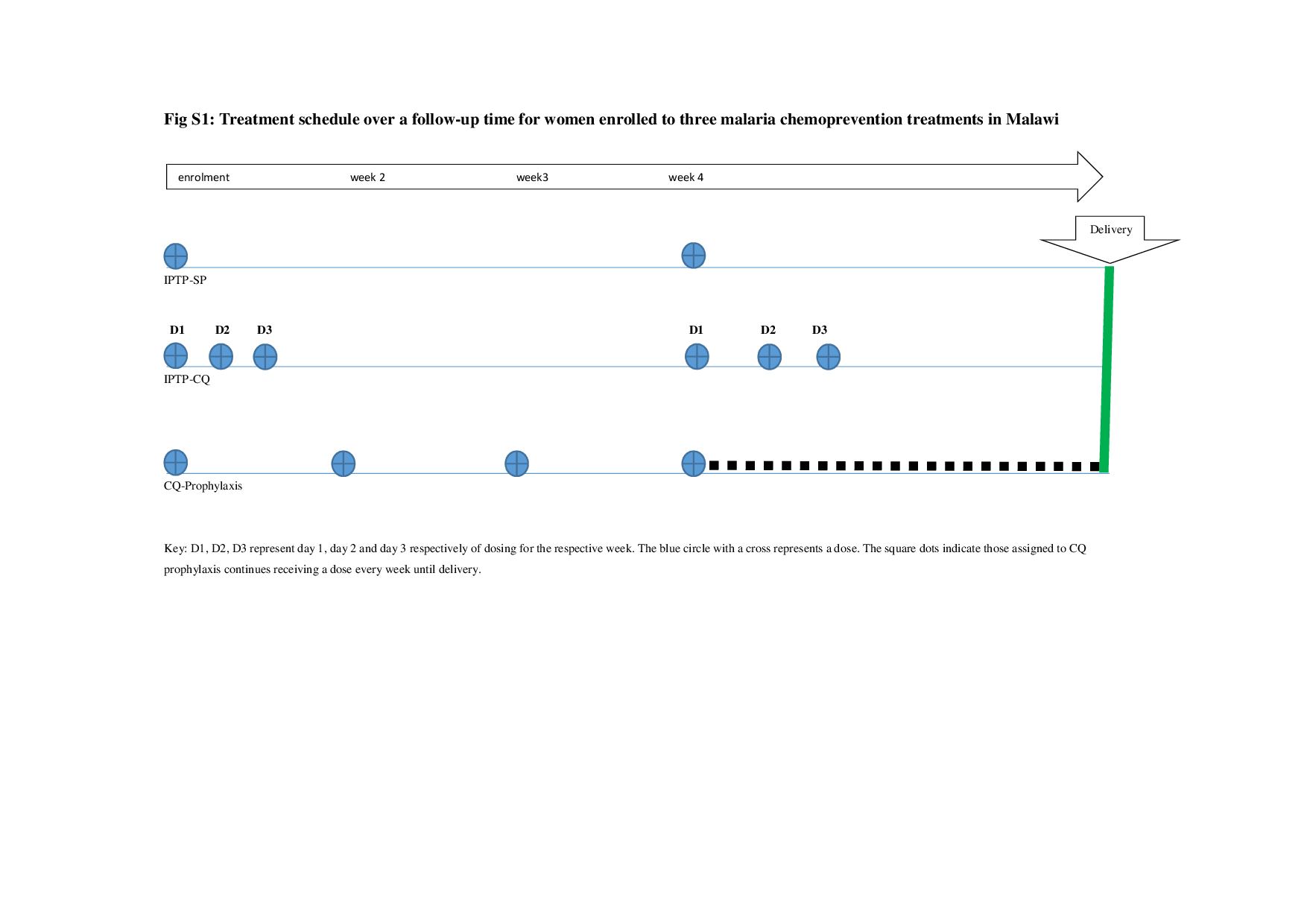

Supplement: S1 Fig — (TIF) [file pone.0262797.s003.tif]
